# The dream of health information for all

**DOI:** 10.12688/f1000research.6950.2

**Published:** 2016-05-24

**Authors:** Alvaro Proaño, Eloy F Ruiz, Ruben Porudominsky, Jose Carlos Tapia

**Affiliations:** 1 Facultad de Medicina “Alberto Hurtado”, Universidad Peruana Cayetano Heredia, Lima, Peru; 2 CONEVID, Unidad de Conocimiento y Evidencia, Universidad Peruana Cayetano Heredia, Lima, Peru

**Keywords:** Publishing, Education, Communication, Peru, Medical Students

## Abstract

In 2004, an influential report in *The Lancet* suggested that open health information for all could be achieved by 2015. Unfortunately, this goal has not yet been accomplished. Despite progress in obtaining quality scientific articles in Latin America, it remains difficult to reliably access new and cutting-edge research. As graduating Peruvian medical students, we have confronted many obstacles in obtaining access to quality and up-to-date information and a constant tension between accessing "what is available" rather than "what we need". As we have learned, these limitations affect not only our own education but also the choices we make in the management of our patients. In the following article, we state our point of view regarding limitations in access to scientific articles in Peru and Latin America.

## Introduction

Since the earliest days of scientific publishing, the dissemination of research has been intended to maximize impact rather than profit ^1^. Although this philosophy remains with the scientific community to this day, for-profit publishers continue to charge for access. However, with the development of the internet, a new hope was born for open access ^2^.

In 2004, Godlee *et al.* proposed in *The Lancet*, that we could achieve health information for all by 2015 ^3^; nevertheless, eleven years later this goal has not yet been fulfilled. Instead, the open medical repository is just a fraction of all the information out there. For example, during 2011, only 17% of the 1.66 million articles published and indexed in Scopus were available through open access ^4^.

Full access to scholarly articles carries several benefits to researchers, healthcare professionals, policymakers and to society in general ^5, 6^. The alternative scenario results in low literacy for medical professionals, affecting the diagnosis and treatment of patients. The scarcity of open literature contributes to an out-of-date medical practice, where health professionals *“[believe] that they can go about their job without new information”* ^7^. This type of practice is unacceptable and should be changed for the well-being of our patients.

Medical school is a key moment to learn how to read critically the current research and to keep up-to-date with it. If we learn it as students, we will carry this practice throughout our career, but if we do not, it will only become harder later. In this context, after having gone through seven years of medical school, we believe it is important to state our point of view, as medical students, regarding the limitation of access to scientific articles in Peru and Latin America.

## Access to research publications in Latin America

Nowadays, without any doubt, the three most visible Latin American efforts to achieve the dream of open access and to catalyze globalized science are the *Literatura Latinoamericana y del Caribe en Ciencias de la Salud* (LILACS), the *Scientific Electronic Library Online* (SciELO), and The RedALyC project ( *Red de Revistas Científicas de América Latina y el Caribe, España y Portugal*) ^8– 11^. LILACS is a database created by Biblioteca Regional de Medicina (BIREME) and the Pan-American Health Organization (PAHO) ^11^. Since 1982, it had become a crucial resource for the identification of Latin America’s peer-reviewed and "grey" literature on medicine, public health and epidemiology ^11^. SciELO was conceived in 1997 as a pilot program to create a virtual library of Brazilian biomedical journals ^8, 9^. This later became an expanding publishing platform for Latin American journals that currently includes 15 countries from Latin America, Europe and Africa ^9^. RedALyC, established in 2003, was born out as a need to cover the social sciences and humanities (not initially covered by SciELO) that also widened its original focus to all disciplines ^8^. Free-full-text articles in this region have prevailed because most of the journals are not-for-profit enterprises maintained by public academic institutions or scientific societies. In contrast to the usual ‘article publishing charge’ model of open access, Latin American journals follow a ‘fee-less-free’ model providing immediate free access to the electronic edition of the journals without charging the author’s institution for submission, processing or publication of the article on behalf of medical societies or associations ^8, 9, 12^. This enables greater visibility for thousands of articles from Latin America ^10^.

Latin America has long been active in promoting the visibility of its scientific production ^9^. This has been reflected in several initiatives such as the Network of Virtual Libraries of the Latin American Council of Social Sciences (CLACSO), the Alliance of Agricultural Information Services (SIDALC), and LATINDEX, all created before the Budapest Open Access Initiative in 2002 ^10^. Significantly, LATINDEX was one of the first referencing systems for the Ibero-American region, indexing journals related to social and medical sciences ^9^. As Vessuri *et al*. stated, *“[these are clearly great examples] of a region that was already exploring the possibility of building national collections of full-text electronic journals in open access”* ^9^. The most recent open access initiative in the region is the Federated Network of Institutional Repositories of Scientific Publications (LA Referencia) ^10^. The aspiration of this regional network is to become the principal gateway through which Latin America expresses its scientific production to the rest of the world ^13^. Since it was established in 2012, this interoperable network of national repository systems (including Argentina, Brazil, Chile, Colombia, Ecuador, El Salvador, México, Peru and Venezuela) has gathered more than 800,000 full-text documents available for all ^10, 13^.

Despite the Latin American contribution towards policies in favor of open access, there is still scarce evidence to guarantee the fulfillment of “ *universal access to essential health-care information*” ^3^. In other words, even though information is free in SciELO and RedALyC, most of the journals indexed by these services are outdated. A recent analysis in the field of Dermatology found that 60% of journals in these databases were not recent, had lower impact factors and published fewer issues per year when compared to non-free journals indexed in MEDLINE/Pubmed ^12^.

Outside of the continent, other international initiatives have also recently been developed. Open science publishing platforms, such as the *Public Library of Science* ( *PLoS*), *BioMed Central* ( *BMC*), *PeerJ* or *F1000Research* ^2, 14^ allow scientists to publish their work in a high quality platform that also make their content freely available. Additionally, since 2005, research-funding agencies, such as the Wellcome Trust (UK) and National Institutes of Health (USA), promote a policy that all funded work should be publicly available and provide funds to cover the costs of publishing in open access journals ^15^. These collaborative strategies are increasingly allowing healthcare professionals from developing countries to gain free access to high quality research.

Moreover, there are other global alliances that have also aimed to diminish the gap between what exists and what is available. HINARI ^16^, for example, is one of the four programs from Research4Life, a public-private partnership of the World Health Organization, whose main goal is to provide *“free or low cost access to academic and professional peer-reviewed content online”* ^17^. HINARI has provided access to more than 13,000 journals to developing countries since it was launched in 2002 and has opened the door to a world of relevant and latest medical information ^5, 17^. However, many top-notch journals are not always accessible through HINARI, which in turn creates a major setback for medical students ^18^.

## Access to research publications in Peru

In Peru, the main sources of scientific information available for students and healthcare professionals are journals indexed in the Latin American databases mentioned above. The access to other international journals, such as those indexed in MEDLINE, is limited by the agreements of each institute. Still, there are two Peruvian journals indexed in MEDLINE and both are characterized by providing articles online free of cost. These journals are the *Revista Peruana de Medicina Experimental y Salud Publica* ( http://bit.do/rpmesp), and the *Revista de Gastroenterología del Perú* ( http://bit.do/revgastrope). All articles from these journals follow a ‘fee-less-free’ model and are deposited in their respective SciELO library, enabling flow of information and greater visibility ^19^.

In addition, Peruvian universities have developed strategies to keep their scientific production visible, available online and free of cost. We will mention three examples of these initiatives. Some universities require students to finish their career with a thesis that is then stored online in the online *CyberTesis* repository. This project was created to allow all theses to stay available online; however, this project is run independently by each school. This is a problem because its not a centralized archive but instead independent local ones. Researchers like to look for information in centralized databases, or at least use a meta-searcher. Local universities should join initiatives to centralize and share their repositories making it easier for researchers to read and cite them. Universities also possess their own independent journals and offer online repositories for the publications found therein. University-run journals can also upload their publications to SciELO repositories, like the *Revista Medica Herediana* ( http://bit.do/revmedhered) from the Universidad Peruana Cayetano Heredia. Finally, as in other parts of the world ^20^, Open Access Week is celebrated locally by some Universities such as Pontificia Universidad Católica del Perú ^21^ and Universidad de Lima ^22^.

The government of Peru also promotes open access. Under the Peruvian Law N° 30035, there must be a national open-access repository managed by the National Council of Science and Technology and Technological Innovation (CONCYTEC) ^23^. This repository provides access to scientific productions developed in our country ( http://alicia.concytec.gob.pe/vufind/) and also grants access to scientific databases such as ScienceDirect, EBSCO and ProQuest through its virtual library ( http://proyectos.concytec.gob.pe/access/). However, this initiative is not well known among students and entails several registration requirements that are difficult for students to fulfill.

## Our perspective

We have completed seven years of medical education in Peru, and as we have passed through each stage of basic science, pre-clinical and clinical work, our need for updated knowledge has increased. This process has a huge impact on our training because medical school is the period during which we are expected to learn to critically read and to keep up-to-date with current clinical research. Our ability to master this skill then reflects on our clinical decision making and ultimately, affects our patients. Therefore, access to information is not only an intellectual exercise, but also an ethical obligation of our profession. If we ever expect to improve the lives of our patients, we need *“up-to-date, easy, fast, reliable, and affordable accesses to scientific information”* ^24^.

In 2007, Villafuerte-Gálvez *et al*. suggested that students in low-income countries have a difficult time maintaining and advancing their knowledge and skills due to the limited access to pay-per-view journals ^18^. This could become a threat to the student’s education since most of these journals require readers to pay a fee at an unattainable cost (USD20–USD45) for the majority of medical students in developing countries ^18^, making access to research a “pay-wall” to overcome ^20^.

Regardless of the efforts aforementioned, we believe that access should have more promotion by local governments and journals. Governments should keep allocating money to help pay open access initiatives and, on the other hand, journals should offer waivers to authors that cannot pay. Some journals already do this, however many do it depending on the country’s income. This becomes a problem when a country changes its classification (eg. from low income country to middle income country), but its researchers are still lacking the resources. This is the case of Peru. Hence, we do not think that waivers should be based solely on the author’s country affiliation, but instead on a case-by-case basis.

It has been shown that at least one in three (33%) recent Peruvian medical graduates use MEDLINE/PubMed to search for scientific articles ^25^ which is much lower than that reported in physicians from USA (81%), Canada (76%) or UK (77%) ^26^. However, we found no data on how many of these queries achieve access to full-text articles. In our experience, *“only through removing the barriers to access to global research that health improvements can be accelerated”* ^27^. The problem we hope to tackle some day is the one related to the *“the ‘last mile’ of the process which actually delivers the document or other source that has been searched for”* ^28^.

In order to obtain the best medical knowledge, as medical students from a developing country, we must overcome two main obstacles: access and language. Even if barriers to access can be overcome, the language barrier may remain. English is considered the language of biomedical science. However, not all scientists know it, especially in rural and developing areas. It is a shame when researchers studying the health problems of a rural area only report their findings in English. How will the healthcare personnel from that area learn and apply this knowledge? Some Latin American journals (ie. Medwave) have aimed to lessen this gap by providing the same article, with the same digital object identifier (DOI), in more than one language. This is obviously extra work for both the publisher and the authors, but if the information cannot be understood, then it will not be applied.

As The Finch Group Report suggests, strategies that aim to achieve the dream of *“Health information for all”* should be guided by four principles: access, usability, quality, and cost and sustainability ^29^. First, research should be freely accessible to individuals and organizations all around the globe. As mentioned earlier, the key issue about this criterion is the availability of free full-text, ideally in the native language where the research was done. Second, usability implies being able to access the full range of the latest tools to organize, analyze and manipulate the content relevant to their work. Third, the rigorous peer-review process must be maintained without affecting the previous principles. The openness and transparency of academic publishers (not-for-profit) guarantee the quality and development of publications supported by peer-review. Finally, citing The Finch Report, “ *the main point of cost and sustainability is that no form of publishing is cost-free, and the key requirement is therefore that publishers, whether commercial or not-for-profit, should be able to generate revenues to meet the cost of services they provide*” ^29^.

Eleven years have passed and despite some initiatives, the goal of open access has not been achieved. We have not lost hope that universal access to essential health-care information will occur, but maybe it is time to change our individual approach and for governments, universities and academic societies to develop a unified strategy by which open access can be achieved, while still fulfilling the aforementioned four principles.

Until then, will we keep dealing with the dilemma of accessing “what is available” rather than “what we need”?
