# Supplementary material for: The dream of health information for all
Source: F1000Res. 2016 May 24;5:40. Originally published 2016 Jan 8. [Version 2] doi: 10.12688/f1000research.6950.2 (PMC4813630; doi:10.12688/f1000research.6950.2)
Supplement: Peer review history

# Peer review: Referee response for version 2

**DOI:** 10.5256/f1000research.9235.r14690

**Published:** 2016-06-29

**Authors:** Lorna Wildgaard

**Affiliations:** 1 Royal School of Library and Information Science(RSLIS), Faculty of Humanities, University of Copenhagen, Copenhagen, Denmark

I have no further comments to this paper (V2). The authors have fully addressed the comments I made to version one and thoroughly revised the article. It is a very nice paper, that I am sure will create a lot of discussion about access to the best evidence.

I have read this submission. I believe that I have an appropriate level of expertise to confirm that it is of an acceptable scientific standard.

# Peer review: Referee response for version 1

**DOI:** 10.5256/f1000research.7485.r12343

**Published:** 2016-03-29

**Authors:** Geoffrey Williams

**Affiliations:** 1 Department of Document Management, University of Southern Brittany, Lorient, France

This is about dreams, and dreams do not always come true, even open access ones.

This is an interesting opinion text, and not a researched article. It therefore aims to stimulate discussion about the key issue of open access, particularly in the medical world. It raises the interesting, and often overlooked, issue as to access to research for medical practitioners and not just researchers.

There are a number of issues. One is the control of the big publishers who have no intention to release archived texts. Whilst new production may come out as gold open access, there is still a wealth of hidden data. As they say, citing the Finch report, there is a question of cost as publishing one has a cost that the researcher must pay. Another issue is one of language, and that is related to evaluation criteria and indicators. As long as international is seen as a proxy for quality, and as long as the English language will be seen as international, there will be a problem. In years to come, their dream will come increasingly true, but to hasten it a change of mindset on language is vital, especially as Spanish is far from a minority language. The big question will remain archives as the publishers are sitting on a gold mine, and they know it.

I have read this submission. I believe that I have an appropriate level of expertise to confirm that it is of an acceptable scientific standard.

# Peer review: Referee response for version 1

**DOI:** 10.5256/f1000research.7485.r11881

**Published:** 2016-03-29

**Authors:** Alfonso Rodriguez-Morales

**Affiliations:** 1 Faculty of Health Sciences, Universidad Tecnológica de Pereira, Pereira, Colombia

This is a very interesting article facing a challenge for developing countries, costs of publications. Whilst Open Access is excellent in this setting because allows wide access for everybody, also implies costs for authors in most journals, which is also difficult. Particularly this should be considered more in this discussion. Goverments and institutions should be more responsible covering those costs. But also journals should be responsible and consider extreme cases of inability to pay, then offering waivers, and particularly giving different costs for authors from developing countries, regardless of the classification of the World Bank, which is not fair. For example, the HINARI initiative has fallen short for this reason. Many countries there, included Colombia and Peru, were considered according to the WB as countries with an income not classifiable to have access. Then, this is unfair. Anyway, I would like to see more of this discussion in the paper.

I have read this submission. I believe that I have an appropriate level of expertise to confirm that it is of an acceptable scientific standard.

# Peer review: Referee response for version 1

**DOI:** 10.5256/f1000research.7485.r12114

**Published:** 2016-02-01

**Authors:** Lorna Wildgaard

**Affiliations:** 1 Royal School of Library and Information Science(RSLIS), Faculty of Humanities, University of Copenhagen, Copenhagen, Denmark

Proaño *et al.* present an interesting opinion paper about the availability of health information for health practitioners in Latin America. The title gains in irony after one reads the article, which concludes that health information for all remains a dream and not an accomplishable goal. The abstract clearly contextualizes the article to the opinions of the authors and adequately encapsulates the depth of the subject matter. The arguments presented in the article are clearly stated as opinions based on personal experiences, and thus are not necessarily substantiated by evidence gathered from relevant literature. I recommend the article be approved as an opinion paper – this is not ground breaking new knowledge - but is a topic that needs discussion as in the end, access to information can affect clinical decisions.

I recommend the following minor revisions:

p.3, column 1, line 18-20.  “This project was created to allow all theses to stay available online: however, this project is run independently by each school.”

Please include a sentence on why this is problematic – is it a meta-data problem, incompleteness, etc. Do the authors have a solution?

p.3, column 2, line 1. “In 2007, Villafuerte-Gálvez *et al. *(as a Peruvian medical student)

Please correct the use of the singular to the plural eg (as Peruvian medical students)

p3. Column 2, line 9-12. “It has been shown that at least one in three recent Peruvian medical graduates use Medline/PubMed….”

Is this surprisingly different from any other country? In the referenced article by Mejia *et al.* (ref.#24) 34% of the sample regularly used Medline/PubMed. Other studies, using other countries, for example by Davis (2011, follow the link below), report use of PubMed/MEDLINE to be between 70 and 84%.

https://www.researchgate.net/profile/Karen_Davies/publication/49763179_Physicians_and_their_use_of_information_A_survey_comparison_between_the_United_States_Canada_and_the_United_Kingdom/links/004635286b7abf103d000000.pdf

p.3, column 2, line 51. “…but maybe is time to change..”

Please correct to “…but maybe it is time to change…”

p4, column 1, line 2. “ …fulfilling the four principles aforementioned.”

Please change to “…fulfilling the aforementioned four principles.”

p.4, column 1, line 3-4.  “Will we keep dealing with the dilemma of accessing “what is available” rather than “what we need”?

The ending could be stronger. Please consider rewriting this final sentence. Try something like: “Until then, we will keep dealing with the dilemma of accessing “what is available” rather than “what we need”.

For my personal interest:

p.3, column 2, line 26-28 “Some Latin American journals have aimed to lessen the gap by providing the same article, with the same digital object identifier, in more than one language”

This is interesting, I would like to learn more. Is this allowed, ethically? Is this not self-plagiarism? How do you cite such publications and do they count as one or two separate works?

I have read this submission. I believe that I have an appropriate level of expertise to confirm that it is of an acceptable scientific standard.

# Response

**Published:** 2016-04-05

**Authors:** Alvaro Proaño

**Affiliations:** Universidad Peruana Cayetano Heredia, Peru

Thank you very much for your suggestions and for taking the time to review our paper. We look forward to any comments you have on this in the future.

Response to specific comments is as follows:

- p.3, column 1, line 18-20.  “This project was created to allow all theses to stay available online: however, this project is run independently by each school.”
  Please include a sentence on why this is problematic – is it a meta-data problem, incompleteness, etc. Do the authors have a solution?
  - **Response**: “This is a problem because its not a centralized archive but instead independent local ones. Researchers like to look for information in centralized databases, or at least use a meta-searcher. Local universities should join initiatives to centralize and share their repositories making it easier for researchers to read and cite them.”
- p.3, column 2, line 1. “In 2007, Villafuerte-Gálvez *et al. *(as a Peruvian medical student)
  Please correct the use of the singular to the plural eg (as Peruvian medical students)
  - **Response**: Change made
- p3. Column 2, line 9-12. “It has been shown that at least one in three recent Peruvian medical graduates use Medline/PubMed….”
  Is this surprisingly different from any other country? In the referenced article by Mejia *et al.* (ref.#24) 34% of the sample regularly used Medline/PubMed. Other studies, using other countries, for example by Davis (2011, follow the link below), report use of PubMed/MEDLINE to be between 70 and 84%.
https://www.researchgate.net/profile/Karen_Davies/publication/49763179_Physicians_and_their_use_of_information_A_survey_comparison_between_the_United_States_Canada_and_the_United_Kingdom/links/004635286b7abf103d000000.pdf
  - **Response**: We agree with your point of view and think that contrasting Peruvian reality with the one in the UK brings about a good comparison for readers to understand Peru’s reality. Thus, it now reads: “It has been shown that at least one in three (33%) recent Peruvian medical graduates use MEDLINE/PubMed to search for scientific articles 24 which is much lower than that reported in physicians from USA (81%), Canada (76%) or UK (77%) add new citation; however, we found no data on how many of these queries achieve access to full-text articles.”
- p.3, column 2, line 51. “…but maybe is time to change..”
  Please correct to “…but maybe it is time to change…”
  - **Response**: Change made
- p4, column 1, line 2. “ …fulfilling the four principles aforementioned.”
  Please change to “…fulfilling the aforementioned four principles.”
  - **Response**: Change made
- p.4, column 1, line 3-4.  “Will we keep dealing with the dilemma of accessing “what is available” rather than “what we need”?

The ending could be stronger. Please consider rewriting this final sentence. Try something like: “Until then, we will keep dealing with the dilemma of accessing “what is available” rather than “what we need”.

- **Response**: Change made

- For my personal interest:
  p.3, column 2, line 26-28 “Some Latin American journals have aimed to lessen the gap by providing the same article, with the same digital object identifier, in more than one language”
  This is interesting, I would like to learn more. Is this allowed, ethically? Is this not self-plagiarism? How do you cite such publications and do they count as one or two separate works?
  - **Response**: This is allowed ethically, because it would have the same MEDLINE/PubMed entry. Thus, we do not believe this is self-plagiarism because it does not count as two publications, but just one (same DOI) with two different ways of viewing it. I think this does not pose a big problem, because readers know that it is the same version in different languages. For example, the journal Medwave provides, within the same Pubmed Abstract page two options: English or Spanish such as in this case: **https://www.ncbi.nlm.nih.gov/pubmed/26248156**
